# Why pathogen genomics is crucial in Africa’s public health

**DOI:** 10.4102/ajlm.v12i1.2166

**Published:** 2023-09-22

**Authors:** Lamech M. Mwapagha

**Affiliations:** 1 Department of Biology, Chemistry and Physics, Faculty of Health, Natural Resources and Applied Sciences, Namibia University of Science and Technology, Windhoek, Namibia

## Introduction

Pathogens are bacteria, parasites, fungi and viruses that cause diseases by evading the body’s immune system. HIV, *Bacillus anthracis*, Ebola virus, Zika virus, severe acute respiratory syndrome coronavirus 2 and mpox virus are some of the pathogens that cause serious diseases. Thus, understanding the genetic information of these pathogens could play a significant role in curbing the spread of infectious diseases. The traditional and standard pathogen detection methods rely on identifying previously known agents associated with a specific disease, with most conventional laboratory assays having low pathogen detection efficiency and involving time-consuming tasks.^1,2^ Despite being considered the gold standard in pathogen identification, these traditional methods are still incapable of detecting causative agents quickly and precisely, delaying infection diagnosis and complicating disease surveillance.^3^ The introduction of next-generation sequencing (NGS) in pathogen identification is critical in preventing and treating infectious diseases by allowing pathogen genomes to be determined rapidly and precisely.^4^ However, for Africa to reap the benefits of pathogen genomics, genomic research findings must be effectively translated into public health benefits.

## Infectious diseases

Infectious diseases remain the leading cause of death in Africa. Diseases such as malaria, HIV/AIDS, cholera, tuberculosis, meningitis, hepatitis, schistosomiasis, lymphatic filariasis, sleeping sickness, Ebola, coronavirus disease 2019 (COVID-19), mpox and other well-known existing, emerging, and re-emerging diseases are causing suffering and mortality to a large population in many developing countries. These have become a significant burden on public health and detrimental to African and global economies.^5^ The recent global COVID-19 pandemic demonstrated how unprepared the world was and how it remains vulnerable for the next major health crisis.^6,7^ Other previously known microorganisms have recently resurfaced (such as Ebola and mpox) and caused widespread outbreaks in various African regions. Disease re-emergence now requires Africa to devise and implement new approaches to dealing with current and future infectious disease outbreaks to benefit public health.

## Why pathogen genomics

As high-throughput NGS technologies improve, genome-sequencing costs significantly fall.^8,9^ High-throughput technologies and bioinformatics can thus shed light on disease transmission, virulence, and antimicrobial resistance. Next-generation sequencing technology has resulted in many new opportunities for public health, allowing researchers to examine pathogen genomes in greater depth. Pathogen genomics is already in use in many public health applications, such as precision infection identification and diagnosis (parasites, HIV and tuberculosis), disease surveillance (COVID-19, influenza, salmonellosis, and extraintestinal illness), antimicrobial resistance monitoring (in *Candida auris* and streptococcal pathogens), outbreak investigations (*Salmonella* spp. and *Mycobacterium tuberculosis*), and in developing and evaluating interventions, such as vaccines (COVID-19 and polio vaccines).

## Translating pathogen genomic data

Clinical NGS has been used in oncology to identify genomic abnormalities linked to cancer, such as indels, copy number variants, and single-nucleotide variants.^10^ Clinical NGS has also led in the identification of biomarkers for cancer diagnosis and treatment, with notable examples being the use of personalised NGS panels for adult acute myeloid leukaemia and lung cancer diagnosis.^11,12^ In pathogen identification and diagnosis, targeted metagenomic NGS panels target pathogens associated with specific diseases, such as respiratory and gastrointestinal ailments.^13^ Genomics data has also played a critical role in detecting unexpected or previously undiagnosed pathogens, tracking evolution, tracing local chains of transmission, and investigating the origins of epidemics such as Ebola, malaria, influenza and COVID-19.^14,15^ These data, in turn, facilitated disease diagnosis and the development of effective treatment for various diseases globally.

Pathogen genomic data is increasingly used in Africa to meet the public health demands for disease surveillance, prevention, and control of new illnesses. The Ebola virus outbreak in West Africa (2013–2016) and the Democratic Republic of the Congo (2018–2020), the 2018 Lassa virus outbreak in Nigeria, the discovery of the severe acute respiratory syndrome coronavirus 2 Omicron variant in Botswana and South Africa, and the epidemiological and evolutionary dynamics of respiratory syncytial virus viral populations in Kilifi, Kenya, are some noteworthy examples.^16^ Thus, pathogen genomic data combined with epidemiological data has played a crucial role in advancing our understanding of transmission dynamics, as well as the efficacy of diagnostic tests and vaccine regimens for preventing future outbreaks on the African continent.

## Obstacles to be considered

Despite the enormous potential of pathogen genomics in addressing public health issues, Africa has yet to capitalise fully on these benefits due to various challenges.^17^ Nonetheless, several factors must be considered before Africa fully implements pathogen genomics. Among these factors are:

- Next-generation sequencing must be cost-effective compared to conventional methods, that is, personalised medicine, laboratory equipment upgrades, computer resources, and training.
- Laboratory and analytical capabilities in clinical and public health systems must be adaptable to enable the rapid development of NGS technologies and platforms.
- Systems should integrate pathogen genome sequences, bioinformatics, and epidemiology to facilitate knowledge exchange on disease surveillance, outbreak, and control.
- Choosing the right sequencing platform and bioinformatics analysis tools is critical, because it depends on sequencing objectives and requires accuracy, efficiency, and cost trade-offs that may not be universally applicable to all infectious diseases and public health laboratories.
- Ethical, legal, and social frameworks addressing access discrimination^18^ to precision medicine, data sharing, protection, and management in collaboration with national, regional, and global partners for infectious disease surveillance, prevention and control.

## Way forward

Pathogen genomics has great potential to improve public health in Africa by allowing for more effective disease surveillance, outbreak containment, and control investigations to inform vaccine development. A number of factors must be considered for Africa to reap these benefits. Firstly, infrastructure must be built and new technologies must be introduced and integrated into existing infrastructure (computational and storage systems) to support the rapid change in genomics technology. Secondly, capacity building to address the scarcity of African scientists with genomic and computational expertise is needed to translate pathogen genomics into public health systems. Thirdly, collaboration with various public health policymakers (national public health institutes, academic institutions, ministries of health, and non-governmental organisations) should be encouraged to strengthen public health research capacity on disease surveillance and control. Fourthly, establish public health biobanks to aid our understanding of genetic susceptibility and support healthcare research strategies for infectious disease surveillance, diagnosis, and prognosis.^19,20,21^ Fifthly, all players should embrace guiding principles and standards for pathogen genomic data sharing that are ethical, egalitarian, efficient, and effective to inform public health policy and response.^8,9,22,23^

Africa is on the right track, having established programmes supporting pathogen genomic initiatives. For example, the Africa Centres for Disease Control and Prevention through the Africa Pathogen Genomics Initiative and the African Academy of Sciences are programmes that promote scientific and technological development in Africa while supporting public health pathogen genomics and bioinformatics. Furthermore, in addition to equipping laboratories with high throughput genomic sequencing equipment, the Africa Pathogen Genomics Initiative is working to improve laboratory staff technical capacity by collaborating with key partners (African Centre of Excellence for Genomics of Infectious Diseases, Kenya Medical Research Institute, International Livestock Research Institute-Kenya, South African National Bioinformatics Institute, National Institute for Communicable Diseases, KwaZulu-Natal Research Innovation and Sequencing Platform/Centre for Epidemic Response & Innovation, Institut Pasteur de Dakar, and Institut National de la Recherche Biomédicale) engaged in genomic sequencing and bioinformatics to facilitate training, knowledge-sharing, and technical support. Other programmes, such as the African Field Epidemiology Network and the African Society for Laboratory Medicine, also strengthen public health laboratory networks and systems throughout Africa by increasing public health capacity, monitoring disease outbreaks, and promoting the One Health approach.

Although advances in NGS have brought us closer to a complete understanding of infectious diseases, much work remains. The undeniable value of NGS, particularly for rapid and precise pathogen identification, can not be overemphasised. If science is to progress, it must delineate the diverse array of microorganism sequences and improve genomic tools and methodologies. Overall, if Africa is to reap the public health benefits of these novel approaches to disease surveillance, prevention, and control, all public health policymakers and global partners (national public health institutes, ministries of health, academic institutions, non-governmental organsiations, industry, government, private sector, philanthropists, and funding agencies) must be involved from the outset of all discussions involving the integration of pathogen genomics into public health benefits.
